# Influence of Carbon Nanotubes on Thermal Stability of Water-Dispersible Nanofibrillar Polyaniline/Nanotube Composite

**DOI:** 10.3390/ma5020327

**Published:** 2012-02-17

**Authors:** Ana López Cabezas, Xianjie Liu, Qiang Chen, Shi-Li Zhang, Li-Rong Zheng, Zhi-Bin Zhang

**Affiliations:** 1 iPack VINN Excellence Center, School of Information and Communication Technology, Royal Institute of Technology (KTH), Stockholm SE-164 40, Sweden; E-Mails: analc@kth.se (A.L.C.); qiangch@kth.se (Q.C.); lirong@kth.se (L.-R.Z.); 2 Department of Physics, Chemistry and Biology, Linköping University, Linköping SE-581 83, Sweden; E-Mail: xjliu@ifm.liu.se; 3 Solid-State Electronics, Department of Engineering Sciences, Uppsala University, P.O. Box 534, Uppsala SE-751 21, Sweden; E-Mail: shili.zhang@angstrom.uu.se

**Keywords:** polyaniline, carbon nanotubes, conducting polymers, thermal stability, Raman spectroscopy

## Abstract

Significant influence on the thermal stability of polyaniline (PANI) in the presence of multi-walled carbon nanotubes (MWCNTs) is reported. By means of *in-situ* rapid mixing approach, water-dispersible nanofibrillar PANI and composites, consisting of MWCNTs uniformly coated with PANI in the state of emeraldine salt, with a well-defined core-shell heterogeneous structure, were prepared. The de-protonation process in PANI occurs at a lower temperature under the presence of MWCNTs on the polyaniline composite upon thermal treatment. However, it is found that the presence of MWCNTs significantly enhances the thermal stability of PANI’s backbone upon exposure to laser irradiation, which can be ascribed to the core-shell heterogeneous structure of the composite of MWCNTs and PANI, and the high thermal conductivity of MWCNTs.

## 1. Introduction

Composite of carbon nanotubes (CNTs) and polyaniline (PANI) represents a new class of carbon-based functional materials which exhibit enhanced properties, for instance, mechanical and electronic aspects due to the presence of MWCNTs and possess similar solubility and processability of PANI [1,2,3,4]. Furthermore, the synergy effect of the two constituents in a composite can lead to novel functions. For instance, PANI in emeraldine base (EB) is insulating with unique optical properties [5] and with the inclusion of CNT, the resulted composite not only keeps the optical properties, but also possesses relatively good conductivity [3]. Therefore, the use of a composite of PANI/CNTs to replace PANI indicates enhanced performance or new functions in the application, such as antistatic coatings, electrochromic coatings, sensors, electroluminescent devices, energy storage, field emission, nonvolatile memory [5]. Compared to the other conducting polymers, PANI has better environmental stability, but its thermal stability is similarly poor when subjected to thermal treatment [6,7], particularly in exposure to light irradiation [8,9]. In this work, we will show that the thermal stability of PANI coated on multi-walled carbon nanotubes (MWCNTs) is significantly influenced. Using light irradiation, the presence of MWCNTs enhances significantly the thermal stability of PANI’s backbone and we will also show that upon thermal treatment, the conversion of PANI from its emeraldine salt (ES) to emeraldine base (EB) form occurs at lower temperature in the presence of nanotubes in the composite.

## 2. Results and Discussion

**Figure 1:**
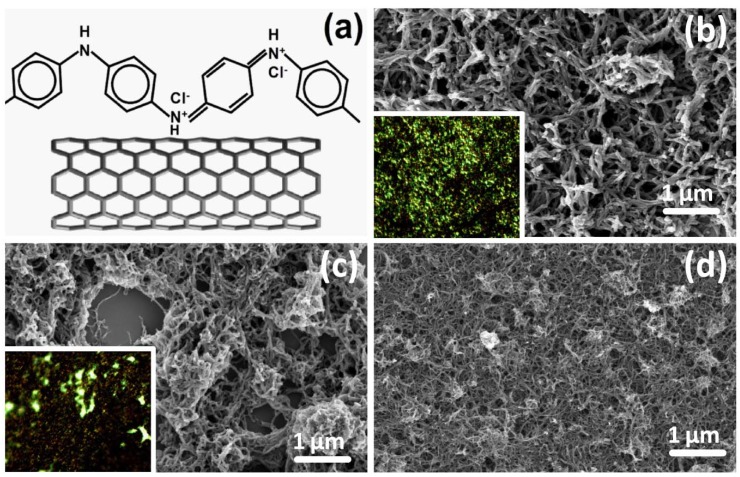
(**a**) Schematic structure of polyaniline in emeraldine base (PANI-ES) and outer-shell of multi-walled carbon nanotube (MWCNT) and high-resolution scanning electron microscopy (HRSEM) image of (**b**) nanofibrillar **(**nf-) PANI; (**c**) nf-PANI/MWCNT-20wt% and (**d**) nf-PANI/MWCNT-50wt%. The insets are the optical microscopy images of the films (nf-PANI (b) and nf-PANI/MWCNT-20% (c)) with 200 × 160 μm.

Figure 1a schematically depicts the molecular structure of PANI-ES attached to MWCNTs, synthesized by means of rapid mixing approach. The result is consistent with the previous report that the polymerization of aniline, with the addition of oxidant, readily occurs on the surface of MWCNTs, possibly due to their role as heterogeneous catalyst and mediator of electron transfer [3]. The as prepared PANI and PANI/MWCNT are highly dispersible in water and thin films can be formed by drop casting technique. By means of high resolution scanning electron microscopy (HRSEM), the nanofibrillar (nf-) morphology is observed across the entire thin film of PANI (Figure 1b) composite with 20% MWCNT content (Figure 1c) and with 50% MWCNT (Figure 1d). In composite, MWCNTs are uniformly coated with PANI observed by transmission electron microscopy (image not shown here). Thereafter, nf-PANI and nf-PANI/MWCNT are used to denote the prepared PANI and composites, respectively. The well-defined core-shell structure of nf-PANI/MWCNT allows the MWCNTs to express excellent solubility and processability in water [10].

**Figure 2:**
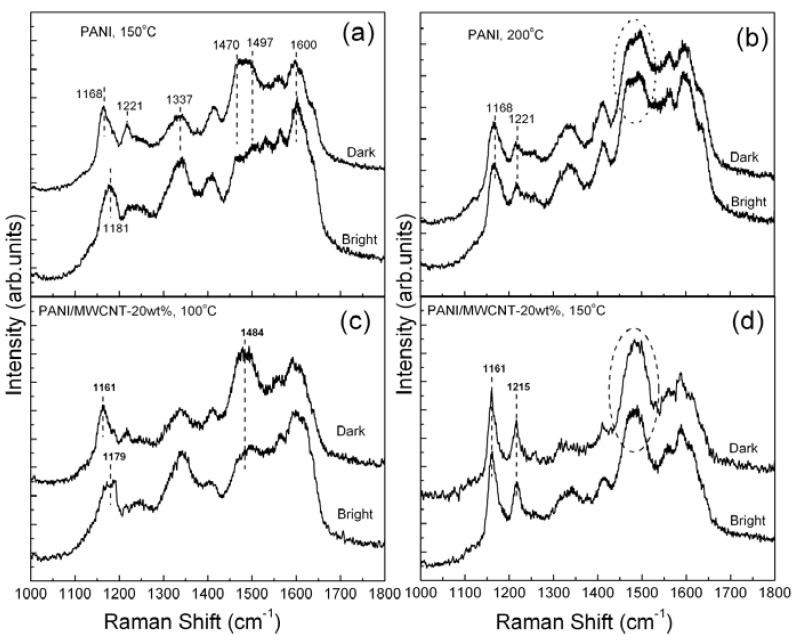
Raman spectra from the bright and dark region of nf-PANI after being baked in air at (**a**) 150 °C in air and (**b**) at 200 °C and those of nf-PANI/MWCNT-20wt% baked at (**c**) 100 °C and (**d**) 150 °C, respectively.

In contrast to the uniformity in nanofibrillar morphology, the thin films of nf-PANI and nf-PANI/MWCNT exhibit an apparent non-uniformity in brightness under optical microscopy as shown in the insets of Figure 1b,c. Raman spectroscopy is used to characterize the possible difference in molecular state in the areas with different optical brightness. The Raman spectra of the nf-PANI obtained intentionally from the bright and dark region show an appreciable difference in intensity of the band from ~1,470 to 1,497 cm^−1^ that is centered at ~1,477 cm^−1^. The difference remains when the thin film underwent baking in air at up to 150 °C (Figure 2a). With the temperature increased to 200 °C (Figure 2b), this band of ~1,477 cm^−1^ from both the bright and dark region is increased and, interestingly, the difference in intensity of this band disappears. In addition, the band of 1,181 cm^−1^ from the bright region, is shifted to the position of the dark region, *i.e.*, 1,168 cm^−1^, when the temperature is increased from 150 °C to 200 °C. In contrast, the band of the dark region remains fixed at 1,168 cm^−1^. Therefore, the Raman spectra of the bright and dark region for nf-PANI essentially become identical after baking the nf-PANI thin film at 200 °C. In the presence of MWCNT, similar changes occur to the PANI except that the temperature at which the Raman spectra from bright and dark region become identical is lower than 150 °C (Figure 2c,d).

Because the band at ~1,477 cm^−1^ is originated from the vibration of quinoid segments of PANI-EB (Table 1), the stronger intensity of the band at ~1,477 cm^−1^ indicates that the dark regions in the pristine thin films may contain a much higher ratio of PANI-EB. The drastic increase of the band at 1,477 cm^−1^ at high temperature (Figure 2b,d) suggests the occurrence of the de-protonation process of PANI-ES, *i.e.*, the conversion of PANI from ES to EB. Although the resonance condition in Raman measurement may slightly depend on the thickness of the nf-PANI and nf-PANI/MWCNT thin film [11], it is hard to explain the distinct difference in intensity of the band at ~1,477 cm^−1^ from the dark and bright regions in Figure 2a. Furthermore, the disappearance of such differences at higher temperatures cannot be interpreted by the effect of difference in film thickness. The difference in the Raman spectra from the thin films of different brightness was once attributed to non-uniformity in microstructure of PANI, e.g., nanofibrillar and flake structure [8]. However, we did not observe any other structural morphology that is different from nanofibrills in the thin films under investigation (Figure 1).

**Table 1:** Assignments of the Raman bands with 514 nm excitation [8,12].

Wavenumber [cm^−1^]	Assignments
1,167	C–H in-plane bending(Q)
1,181	C–H in-plane bending(B)
1,221	C–N stretching (B)
1,477	C=N and CH=CH stretching (Q)
1,600	C=C ring stretching (Q)

**Figure 3:**
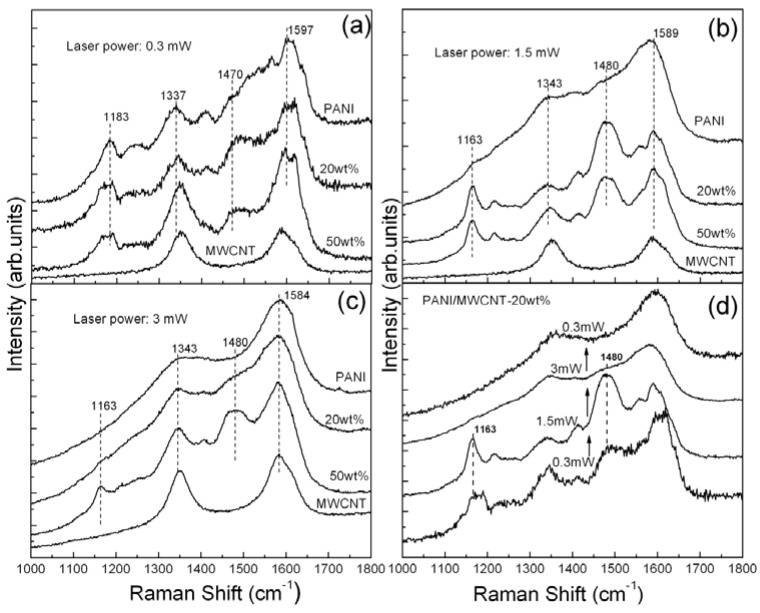
Raman spectra of nf-PANI, nf-PANI/MWCNT-20wt%, nf-PANI/MWCNT-50wt%, and MWCNT collected with 514 nm wavelength laser line at different powers of (**a**) 0.3; (**b**) 1.5 and (**c**) 3mW, respectively; (**d**) Raman spectra of nf-PANI/MWCNT-20wt% obtained on a fixed spot with variable laser power from 0.3 mW to 3 mW and subsequently back to 0.3 mW.

Figure 3 shows the Raman spectra of the nf-PANI and nf-PANI/MWCNT-20wt% and -50wt% obtained from the bright regions using different laser power. When the laser power is low, *i.e.*, 0.3 mW, the Raman spectra of the nf-PANI and nf-PANI/MWCNT reflect the characteristic features of PANI-ES. Compared with the nf-PANI, the nf-PANI/MWCNT shows higher intensity of the band at ~1,477 cm^−1^, the signal from the quinoid segments. When the laser power is increased to 1.5 mW (Figure 3b), the characteristic signals of nf-PANI become almost invisible, indicating that the PANI backbone is nearly destroyed. In contrast, the characteristic Raman bands are clearly present for nf-PANI/MWCNT-20wt% and -50wt%. At the same time, the local annealing by the laser irradiation causes occurrence of de-protonation in nf-PANI/MWCNT. This is reflected by the substantially increased intensity of the band at 1,480 cm^−1^ [9]. With the laser power further increased to 3 mW (Figure 3c), the characteristic Raman signals of nf-PANI completely disappear, leaving the broad band at 1,343 and 1,584 cm^−1^. Meanwhile, the signals from nf-PANI/MWCNT-20wt% similarly become almost invisible. However, the characteristic peaks of PANI persistently remain for nf-PANI/MWCNT-50wt%. As mentioned earlier, the substantial decrease in intensity and subsequent disappearance of all the characteristic features under the local laser irradiation can be attributed to the destruction of PANI backbone. This can be confirmed by the result in Figure 3d which was obtained upon a fixed spot. The PANI-ES in the presence of 20 wt% of MWCNT undergoes de-protonation with laser power raised from 0.3 MW to 1.5 mW and is disintegrated at 3 mW since it loses its characteristic Raman signals. The characteristic Raman signals are not recovered after the laser power is subsequently decreased to 0.3 mW. Therefore, the presence of MWCNT significantly enhances the structural stability of the surrounding PANI upon the exposure to laser irradiation in contrast to the PANI without MWCNT. Such enhancement is more significant with the increase of MWCNT content.

**Figure 4:**
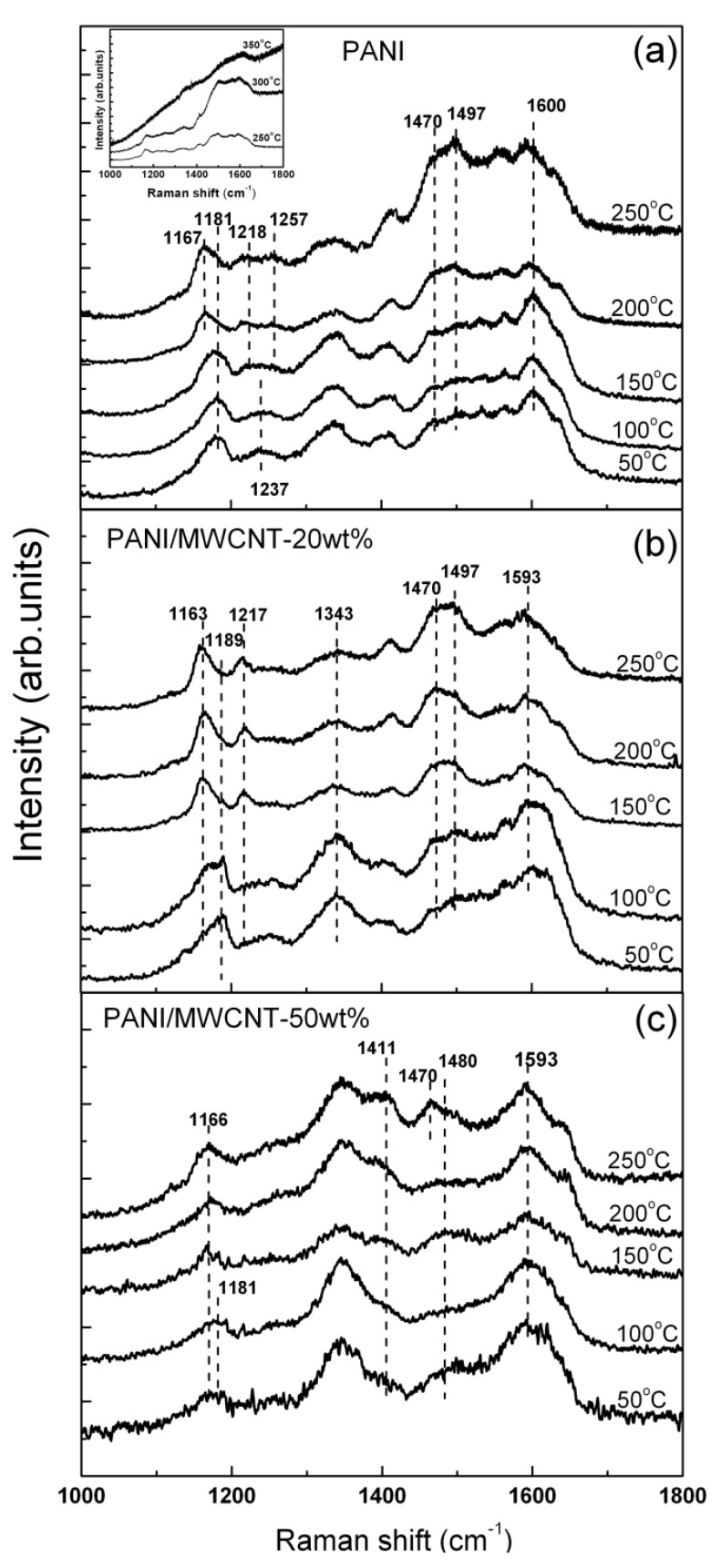
Temperature dependence Raman spectra of (**a**) nf-PANI; (**b**) nf-PANI/MWCNT-20wt%; and (**c**) nf-PANI/MWCNT-50wt% in which the thin films were baked in air before Raman measurement.

As compared to the local laser annealing, the enhancement in structural stability of PANI in the presence of MWCNT is not significant under even thermal treatment in air. Figure 4 illustrates the evolution of the Raman signals from nf-PANI and nf-PANI/MWCNT in ES form obtained from bright regions as a function of temperature.

For nf-PANI in Figure 4a, the broad band from 1,470 to 1,600 cm^−1^ is increased in intensity when the temperature is increased from 200 to 250 °C. With further increase of temperature up to 350 °C, the characteristic Raman features of PANI structure disappear (inset of Figure 4a). For nf-PANI/MWCNT (Figure 4b,c), the appreciable change in the relative intensity of the broad band from 1,470 to 1,600 cm^−1^ occurs between 250 and 300 °C. The nf-PANI/MWCNT with 20 wt% and 50 wt% lose their characteristic Raman features at the same temperature as nf-PANI without MWCNT. If we assume that the increase of broad band from 1,470 to 1,600 cm^−1^ might be due to the occurrence of cross linking of polymer which characteristic signals can be detected with near-IR excitation [9], the presence of MWCNT slightly increase the temperature at which the cross linking of PANI starts to occur. The disappearance of the characteristic peaks at high temperature apparently indicates the destruction of the backbone of PANI.

Although the interaction between PANI and MWCNT is of physical nature, electron transfer can easily occur which was inferred from the enhanced polymerization rate of PANI in the presence of MWCNT as electron transfer mediator [3]. This interfacial electron transfer between PANI and MWCNTs likely facilitates the conversion of PANI from its EB to ES form, as we observed in this work. It is supposed that the mechanisms behind the significant enhancement in thermal stability of PANI’s backbone in nf-PANI/MCWNT composite upon the exposure to laser irradiation lie in the core-shell heterogeneous structure of the composite of MWCNT coated with PANI and the ultrahigh thermal conductivity of MWCNT. The core-shell heterogeneous structure of composite facilitates heat dissipation from PANI resulted from the large intimate contact area between MWCNTs and PANI. Under the resonant excitation of PANI upon the exposure to laser irradiation, the electron transfer between MWCNT and PANI can be enhanced and this might improve dissipating heat from the PANI to the MWCNTs. Due to the ultrahigh thermal conductivity of MWCNT thin film at the level of 3 × 10^3^ W/mK [13], the heat dissipation is expected to be high and therefore the PANI coating could be cooled down rapidly before its backbone is destroyed. With higher content of MWCNT, the PANI coating around MWCNT is smaller in thickness and hence a higher intimate contact area of PANI per volume with MWCNTs is expected. These may result in higher efficiency in heat dissipation from the PANI coating. In the meantime, a higher content of MWCNTs in composite creates larger density of MWCNT percolated network which leads to faster heat dissipation and thus might be responsible for the much more significant enhancement in the stability of PANI’s backbone during local laser irradiation.

## 3. Experimental Section

Pristine high-purity MWCNTs (>90%), aniline, and ammonium persulfate (APS), used as received, were purchased from Sigma–Aldrich Co. The diameters of the outer wall and inner wall of MWCNTs are 10–15 nm and 2–6 nm, respectively, and the average length of them is 0.1–10 μm.

PANI was prepared by chemical oxidation polymerization of aniline with APS and the composites of PANI/MWCNT were synthesized by in-situ polymerization of PANI in the presence of MWNT. The composites were synthesized with different percentage in weight of nanotubes respect to aniline monomer, namely, 2, 20 and 50 wt%, respectively. In a typical reaction procedure, the appropriate amount of nanotubes was sonicated for 3 h in 12.5 mL HCl 1M in order to disperse them in the acidic solution. Aniline monomer was then added to the suspension and shaken for 3min and left still for 30 min. After that time, a 12.5 mL HCL 1M containing APS (aniline:APS molar ration 1:1) was added at once to the aniline solution and shaken for 30 s. The mixture was then left still for 2 h at room temperature. The resulting composite was filtrated and washed with deionized water and methanol.

Stable water dispersions of PANI and composites were prepared by short ultracentrifugation process. The pH of all the dispersions was adjusted to pH = 3 with 0.1 M HCl solution in order to dope polyaniline. Thin films of these materials were prepared by drop casting of the stable dispersions onto a clean oxidized Si wafer and let dry at 50 °C for several hours.

The films were heated on a conventional hot plate in air atmosphere at 100 °C, 150 °C, 200 °C, 250 °C, 300 °C and 350 °C for 3 h and let them cool down before taking Raman measurement.

The morphologies of the pure polyaniline and the resulting composites were characterized using a high resolution field emission transmission electron microscopy (Jeol HR FEGTEM 2100F). The molecular structure and thermal behavior of PANI and composites were studied by Raman spectroscopy with a 514 nm wavelength Argon laser. Raman measurement was conducted on at least 3 spots which were randomly chosen for each sample. During Raman measurement, the objective was set to 50× and the acquisition of data was 100 s.

## 4. Conclusions

Water-dispersible nanofibrillar composites of MWCNT fully coated with PANI in ES, formed with different MWCNT content, were prepared by means of rapid mixing polymerization. The thin films prepared by drop casting of the suspensions exhibit non-uniformity in molecular states. The molecular state of the thin films becomes uniform upon thermal treatment. The presence of MWCNT facilitates the occurrence of de-protonation process in PANI upon thermal treatment, and significantly enhances the thermal stability of PANI backbone upon laser irradiation. The improvement in structural stability of PANI’s backbone in composite can be explained by efficient heat dissipation from the PANI coating around MWCNT, resulting from the large contact area between MWCNT and PANI, due to the core-shell heterogeneous structure of the composite and the high thermal conductivity of MWCNTs.
